# 2-(4-Chloro­benzo­yl)-1-(diamino­methyl­ene)hydrazinium chloride monohydrate

**DOI:** 10.1107/S1600536810014108

**Published:** 2010-04-24

**Authors:** V. M. Chernyshev, A. V. Chernysheva, E. V. Tarasova, V. V. Ivanov, Z. A. Starikova

**Affiliations:** a South-Russia State Technical University, 346428 Novocherkassk, Russian Federation; b A. N. Nesmeyanov Institute of Organoelement Compounds, 119991 Moscow, Russian Federation

## Abstract

In the cation of the title compound, C_8_H_10_ClN_4_O^+^·Cl^−^·H_2_O, the guanidinium group is planar (maximum deviation = 0.0001 Å) and nearly perpendicular to carboxamide group, making a dihedral angle of 87.0 (3)°. The N atoms of the guanidine fragment have a planar trigonal configuration and the N atom of the carboxamide group adopts a pyramidal configuration. In the crystal structure, inter­molecular N—H⋯O, N—H⋯Cl and O—H⋯Cl hydrogen bonds link the cations, anions and water mol­ecules into layers parallel to the *bc* plane.

## Related literature

For a related structure, see: Kolev & Petrova (2003 ▶). For amino­guanidine structures, see: Bharatam *et al.* (2004 ▶); Koskinen *et al.* (1997 ▶); Hammerl *et al.* (2005 ▶); Macháčková *et al.* (2007 ▶); Murugavel *et al.* (2009*a* ▶,*b* ▶). For the preparation of guanyl hydrazides, see: Grinstein & Chipen (1961 ▶). For the application of guanyl hydrazides in the synthesis of 3-substituted 5-amino-1,2,4-triazoles, see: Dolzhenko *et al.* (2009 ▶).
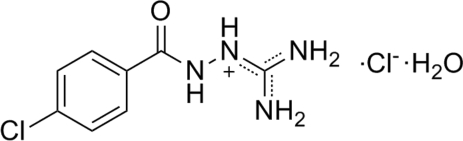


## Experimental

### 

#### Crystal data

C_8_H_10_ClN_4_O^+^·Cl^−^·H_2_O
*M* *_r_* = 267.12
Monoclinic, 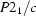

*a* = 19.349 (4) Å
*b* = 4.3563 (9) Å
*c* = 14.516 (3) Å
β = 102.360 (3)°
*V* = 1195.2 (4) Å^3^
*Z* = 4
Mo *K*α radiation
μ = 0.54 mm^−1^
*T* = 100 K
0.40 × 0.30 × 0.15 mm

#### Data collection

Bruker APEXII CCD area-detector diffractometer
Absorption correction: multi-scan (*SADABS*; Bruker, 2004 ▶) *T*_min_ = 0.814, *T*_max_ = 0.924
9756 measured reflections
2330 independent reflections
2099 reflections with *I* > 2σ(*I*)
*R*_int_ = 0.034

#### Refinement

*R*[*F* ^2^ > 2σ(*F* ^2^)] = 0.063
*wR*(*F* ^2^) = 0.142
*S* = 1.17
2330 reflections
145 parameters
H-atom parameters constrained
Δρ_max_ = 0.79 e Å^−3^
Δρ_min_ = −0.41 e Å^−3^

### 

Data collection: *APEX2* (Bruker, 2004 ▶); cell refinement: *SAINT* (Bruker, 2004 ▶); data reduction: *SAINT* and *XPREP* (Bruker, 2005 ▶); program(s) used to solve structure: *SHELXS97* (Sheldrick, 2008 ▶); program(s) used to refine structure: *SHELXL97* (Sheldrick, 2008 ▶); molecular graphics: *Mercury* (Macrae *et al.*, 2006 ▶); software used to prepare material for publication: *SHELXTL* (Sheldrick, 2008 ▶), *publCIF* (Westrip, 2010 ▶) and *PLATON* (Spek, 2009 ▶).

## Supplementary Material

Crystal structure: contains datablocks I, global. DOI: 10.1107/S1600536810014108/cv2710sup1.cif

Structure factors: contains datablocks I. DOI: 10.1107/S1600536810014108/cv2710Isup2.hkl

Additional supplementary materials:  crystallographic information; 3D view; checkCIF report

## Figures and Tables

**Table 1:** Hydrogen-bond geometry (Å, °)

*D*—H⋯*A*	*D*—H	H⋯*A*	*D*⋯*A*	*D*—H⋯*A*
N1—H1⋯Cl2^i^	0.90	2.36	3.194 (4)	154
N2—H2⋯O1*W*	0.90	2.24	3.031 (4)	146
N2—H2⋯Cl2^ii^	0.90	2.71	3.260 (4)	121
N3—H3*B*⋯O1^iii^	0.90	1.96	2.848 (3)	167
N3—H3*A*⋯Cl2^iv^	0.90	2.43	3.280 (4)	157
N4—H4*B*⋯O1*W*	0.90	2.04	2.834 (4)	147
N4—H4*A*⋯Cl2^iv^	0.90	2.44	3.286 (4)	156
O1*W*—H1*W*⋯Cl2^v^	0.85	2.58	3.292 (4)	142
O1*W*—H2*W*⋯Cl2	0.85	2.30	3.134 (4)	164

Symmetry codes: (i) 
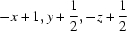
; (ii) 
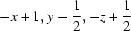
; (iii) 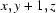; (iv) 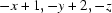; (v) 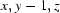.

**Fig. 1.**
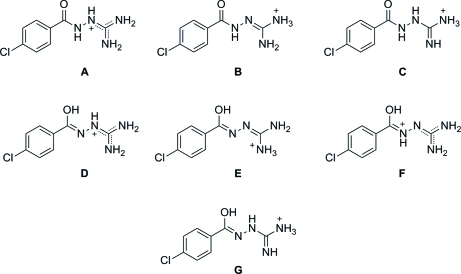
Possible tautomeric forms for 2-guanylhydrazide of 4-chlorobenzoic acid.

**Fig. 2.**
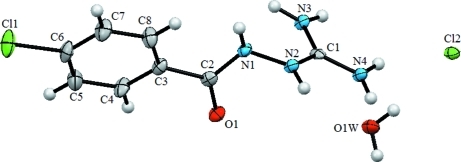
The molecular structure of 4-chlorobenzoic acid 2-guanyl hydrazide hydrochloride monohydrate with the atomic numbering scheme. Displacement ellipsoids are drawn at the 50% probability level.

**Fig. 3.**
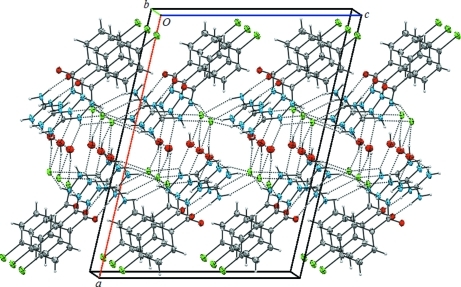
Molecular packing in the crystal, viewed down the b axis. Hydrogen bonds are shown as dashed lines.

## supplementary crystallographic information

### Comment

Carboxylic acids guanyl hydrazides are important starting compounds for the preparation of 3-substituted 5-amino-1,2,4-triazoles (Dolzhenko **et al.**, 2009). Until the present time, the crystal structure of guanyl hydrazides was investigated only for the zwitterionic 2-guanyl hydrazide of carbonic acid (Kolev & Petrova, 2003), which previously was considered as aminoguanidine hydrogen carbonate. Here we report the crystal structure of the title compound.

Carboxylic acids guanyl hydrazides can be regarded as acylated aminoguanidines. Therefore, by analogy with protonated aminoguanidine, it is possible to assume the existence of tautomeric forms A-C (Fig. 1) for the title compound. In addition, the presence of acyl group makes it possible of tautomers D—G, the B—G forms can exist as *cis*- and *trans*-isomers. Quantum chemical calculations predict the tautomer A is to be the more stable for aminoguanidine (Bharatam *et al.*, 2004). This prediction is corroborated by X-ray analyses of aminoguanidine salts (Hammerl *et al.*, 2005; Koskinen *et al.*, 1997; Macháčková *et al.*, 2007; Murugavel *et al.*, 2009a,b).

According to our X-ray investigation, the 4-chlorobenzoic acid 2-guanyl hydrazide hydrochloride monohydrate in the crystal exists as tautomer A (Fig. 2), similarly to aminoguanidine salts. Guanidine fragment (N2/N3/N4/C1) of the molecule is planar. The N1 atom has a trigonal-pyramidal configuration (the sum of bond angles centered on the N1 atom is 354.9° and deviates from the guanidine plane by 0.181 (6) Å. In accordance with the structure of carbonic acid 2-guanylhydrazide (Kolev & Petrova, 2003), carbonyl group is almost perpendicular to the plane of guanidine fragment (dihedral angle between the guanidine and O1/C2/N1 planes amounts 87.0 (3)°). The bonds C1–N3 and C1–N4 have lengths of 1.321 (5) and 1.324 (5) Å, respectively, close to the analogous bonds in aminoguanidine cation, though the C1–N2 bond is somewhat longer – 1.343 (5) Å instead of 1.325-1.341 Å (Hammerl *et al.*, 2005; Koskinen *et al.*, 1997; Macháčková *et al.*, 2007; Murugavel *et al.*, 2009a,b). Apparently, it indicates decrease of π-electron delocalization in the guanidine fragment of the studied molecule in relation to the aminoguanidine and guanidine cations (Bharatam *et al.*, 2004). The N1–N2 bond length of 1.379 (4) Å is essentially equal to the length of analogous bond in the zwitterionic 2-guanyl hydrazide of carbonic acid (1.382 (1) Å, Kolev & Petrova, 2003) and slightly shorter than in aminoguanidine salts (1.396-1.414 Å) (Hammerl *et al.*, 2005; Koskinen *et al.*, 1997; Macháčková *et al.*, 2007; Murugavel *et al.*, 2009a,b). The negative inductive effect of carbonyl group and decrease in π-electron delocalization result in considerable reduction of basicity of the 4-chlorobenzoic acid 2-guanyl hydrazide in comparison with the aminoguanidine. Thus, we obtained the p*K_a_* = 7.85±0.04 by potentiometric titration of the title compound with 0.1 M aqueous potassium hydroxide, whereas the p*K_a_* = 11.5±0.1 was reported for the aminoguanidine (Koskinen *et al.*, 1997).

The crystal packing is shown in Fig. 3. The C_8_H_10_ClN_4_O cations form stacks along the *b* axis of the monoclinic cell. In the neighbouring stacks along the *c* axis the cations are related by a glide-reflection plane which is perpendicular to [0, 1, 0] with glide component [0, 0, 1/2]). Along the *a* axis the C_8_H_10_ClN_4_O cations of the neighbouring stacks are turned from each other by 180° and displaced on 0.5 of cell parameter in direction of the *b *axis, i.e. they are space related by the 2-fold screw axes with direction [0, 1, 0] at 0, *y*, 1/4 with screw component [0, 1/2, 0]. In the stacks the adjacent cations are connected with each other by the N3—H3B···O1^iii^ hydrogen bonds. The rows of chloride anions and water molecules are localized between the stacks of C_8_H_10_ClN_4_O cations close to the guanidine fragments. Cloride anions additionally stabilize the location of C_8_H_10_ClN_4_O cations in the stacks by means of two groups of hydrogen bonds (Table 1): the N1—H1···Cl2^i^ and N2—H2···Cl2^ii^, the N3—H3A···Cl2^iv^ and N4—H4A···Cl2^iv^. As a result, equally oriented stacks of the cations form layers along the *c* axis with identity period equal to the unit cell parameter *c*. The rows of water molecules are ordered along the *b* axis by means of the hydrogen bonds N2—H2···O1W, N4—H4B···O1W, O1W—H1W···Cl2^v^ and O1W—H2W···Cl2. Thereby, the C_8_H_10_ClN_4_O cations, water molecules and chloride anions form a rigid three-dimensional framework in the crystal.

### Experimental

4-Chlorobenzoic acid 2-guanyl hydrazide hydrochloride monohydrate was prepared by fusion of 4-chlorobenzoyl chloride with aminoguanidine hydrochloride according to Grinstein & Chipen (1961). The crystals suitable for crystallographic analysis were grown by recrystallization from water-ethanol 1:1 mixture.

### Refinement

C-bound H atoms were positioned geometrically (C—H 0.93 Å), while the rest H atoms were located on difference map and further placed in idealized positions (N—H 0.90 Å, O—H 0.85 Å). All H atoms were refined as riding on their parent atoms, with U_iso_(H) = 1.2-1.5 U_eq_(parent atom).

### Crystal data

**Table:**

C_8_H_10_ClN_4_O^+^·Cl^−^·H_2_O	*F*(000) = 552
*M**_r_* = 267.12	*D*_x_ = 1.484 Mg m^−^^3^
Monoclinic, *P*2_1_/*c*	Mo *K*α radiation, λ = 0.71073 Å
Hall symbol: -P 2ybc	Cell parameters from 195 reflections
*a* = 19.349 (4) Å	θ = 3–25°
*b* = 4.3563 (9) Å	µ = 0.54 mm^−^^1^
*c* = 14.516 (3) Å	*T* = 100 K
β = 102.360 (3)°	Plate, colourless
*V* = 1195.2 (4) Å^3^	0.40 × 0.30 × 0.15 mm
*Z* = 4	

### Data collection

**Table:**

Bruker APEXII CCD area-detector diffractometer	2330 independent reflections
Radiation source: fine-focus sealed tube	2099 reflections with *I* > 2σ(*I*)
graphite	*R*_int_ = 0.034
ω scans	θ_max_ = 26.0°, θ_min_ = 2.2°
Absorption correction: multi-scan (*SADABS*; Bruker, 2004)	*h* = −23→23
*T*_min_ = 0.814, *T*_max_ = 0.924	*k* = −5→5
9756 measured reflections	*l* = −17→17

### Refinement

**Table:**

Refinement on *F*^2^	Primary atom site location: structure-invariant direct methods
Least-squares matrix: full	Secondary atom site location: difference Fourier map
*R*[*F*^2^ > 2σ(*F*^2^)] = 0.063	Hydrogen site location: difference Fourier map
*wR*(*F*^2^) = 0.142	H-atom parameters constrained
*S* = 1.17	*w* = 1/[σ^2^(*F*_o_^2^) + (0.*P*)^2^ + 7.6548*P*] where *P* = (*F*_o_^2^ + 2*F*_c_^2^)/3
2330 reflections	(Δ/σ)_max_ < 0.001
145 parameters	Δρ_max_ = 0.79 e Å^−^^3^
0 restraints	Δρ_min_ = −0.41 e Å^−^^3^

### Special details

**Table:**

Geometry. All esds (except the esd in the dihedral angle between two l.s. planes) are estimated using the full covariance matrix. The cell esds are taken into account individually in the estimation of esds in distances, angles and torsion angles; correlations between esds in cell parameters are only used when they are defined by crystal symmetry. An approximate (isotropic) treatment of cell esds is used for estimating esds involving l.s. planes.
Refinement. Refinement of *F*^2^ against ALL reflections. The weighted *R*-factor wR and goodness of fit *S* are based on *F*^2^, conventional *R*-factors *R* are based on *F*, with *F* set to zero for negative *F*^2^. The threshold expression of *F*^2^ > σ(*F*^2^) is used only for calculating *R*-factors(gt) etc. and is not relevant to the choice of reflections for refinement. *R*-factors based on *F*^2^ are statistically about twice as large as those based on *F*, and *R*-factors based on ALL data will be even larger.

### Fractional atomic coordinates and isotropic or equivalent isotropic displacement parameters (Å^2^)

**Table:**

	*x*	*y*	*z*	*U*_iso_*/*U*_eq_	
Cl1	0.04674 (6)	0.7630 (4)	0.43499 (8)	0.0378 (3)	
Cl2	0.61291 (5)	0.8080 (2)	0.16436 (7)	0.0176 (2)	
O1	0.23490 (15)	0.4393 (7)	0.1141 (2)	0.0206 (6)	
N1	0.30500 (17)	0.8203 (8)	0.1875 (2)	0.0167 (7)	
H1	0.3168	0.9410	0.2387	0.020*	
N2	0.36010 (18)	0.7335 (8)	0.1466 (2)	0.0168 (7)	
H2	0.3904	0.5813	0.1693	0.020*	
N3	0.32072 (18)	1.0675 (8)	0.0221 (2)	0.0170 (7)	
H3B	0.2901	1.1579	0.0524	0.020*	
H3A	0.3270	1.1318	−0.0344	0.020*	
N4	0.41145 (18)	0.7296 (8)	0.0186 (2)	0.0191 (8)	
H4B	0.4390	0.5690	0.0416	0.023*	
H4A	0.4156	0.8225	−0.0354	0.023*	
C1	0.3636 (2)	0.8462 (9)	0.0616 (3)	0.0151 (8)	
C2	0.2468 (2)	0.6400 (10)	0.1752 (3)	0.0167 (8)	
C3	0.1974 (2)	0.6938 (10)	0.2392 (3)	0.0189 (9)	
C4	0.1303 (2)	0.5694 (12)	0.2146 (3)	0.0255 (10)	
H4	0.1166	0.4678	0.1573	0.031*	
C5	0.0832 (2)	0.5933 (12)	0.2738 (3)	0.0288 (11)	
H5	0.0379	0.5112	0.2564	0.035*	
C6	0.1048 (2)	0.7415 (12)	0.3591 (3)	0.0248 (10)	
C7	0.1710 (2)	0.8685 (12)	0.3853 (3)	0.0294 (11)	
H7	0.1843	0.9706	0.4426	0.035*	
C8	0.2176 (2)	0.8438 (12)	0.3260 (3)	0.0253 (10)	
H8	0.2627	0.9273	0.3437	0.030*	
O1W	0.48997 (16)	0.3347 (7)	0.1577 (2)	0.0256 (7)	
H1W	0.5036	0.1484	0.1625	0.038*	
H2W	0.5283	0.4382	0.1677	0.038*	

### Atomic displacement parameters (Å^2^)

**Table:**

	*U*^11^	*U*^22^	*U*^33^	*U*^12^	*U*^13^	*U*^23^
Cl1	0.0308 (6)	0.0615 (9)	0.0261 (6)	0.0026 (6)	0.0171 (5)	−0.0009 (6)
Cl2	0.0268 (5)	0.0130 (4)	0.0146 (5)	0.0001 (4)	0.0079 (4)	0.0004 (4)
O1	0.0231 (15)	0.0225 (16)	0.0159 (14)	0.0020 (13)	0.0038 (11)	−0.0030 (13)
N1	0.0226 (17)	0.0160 (17)	0.0134 (16)	0.0018 (15)	0.0078 (13)	−0.0013 (14)
N2	0.0253 (18)	0.0142 (17)	0.0126 (16)	0.0030 (14)	0.0079 (13)	0.0017 (13)
N3	0.0267 (18)	0.0169 (17)	0.0094 (15)	0.0041 (15)	0.0083 (13)	0.0026 (14)
N4	0.0297 (19)	0.0139 (17)	0.0172 (17)	0.0031 (15)	0.0125 (14)	0.0020 (14)
C1	0.022 (2)	0.0111 (19)	0.0118 (18)	−0.0043 (16)	0.0037 (15)	−0.0046 (15)
C2	0.023 (2)	0.017 (2)	0.0103 (18)	0.0039 (17)	0.0025 (15)	0.0039 (16)
C3	0.023 (2)	0.022 (2)	0.0126 (19)	0.0048 (18)	0.0052 (16)	0.0013 (17)
C4	0.024 (2)	0.035 (3)	0.017 (2)	0.000 (2)	0.0056 (17)	−0.011 (2)
C5	0.022 (2)	0.038 (3)	0.029 (2)	−0.002 (2)	0.0102 (18)	−0.002 (2)
C6	0.024 (2)	0.037 (3)	0.016 (2)	0.007 (2)	0.0109 (17)	0.0046 (19)
C7	0.032 (2)	0.042 (3)	0.016 (2)	0.001 (2)	0.0092 (18)	−0.006 (2)
C8	0.024 (2)	0.037 (3)	0.016 (2)	−0.004 (2)	0.0059 (17)	−0.0025 (19)
O1W	0.0256 (16)	0.0157 (15)	0.0359 (18)	0.0004 (13)	0.0076 (13)	0.0033 (14)

### Geometric parameters (Å, °)

**Table:**

Cl1—C6	1.736 (4)	C2—C3	1.488 (5)
O1—C2	1.232 (5)	C3—C4	1.381 (6)
N1—C2	1.352 (5)	C3—C8	1.398 (6)
N1—N2	1.379 (4)	C4—C5	1.384 (6)
N1—H1	0.8999	C4—H4	0.9300
N2—C1	1.343 (5)	C5—C6	1.380 (7)
N2—H2	0.9001	C5—H5	0.9300
N3—C1	1.321 (5)	C6—C7	1.373 (7)
N3—H3B	0.9002	C7—C8	1.377 (6)
N3—H3A	0.9000	C7—H7	0.9300
N4—C1	1.324 (5)	C8—H8	0.9300
N4—H4B	0.8999	O1W—H1W	0.8517
N4—H4A	0.9002	O1W—H2W	0.8542
			
C2—N1—N2	118.8 (3)	C4—C3—C2	118.1 (4)
C2—N1—H1	120.3	C8—C3—C2	122.8 (4)
N2—N1—H1	115.6	C3—C4—C5	121.2 (4)
C1—N2—N1	119.4 (3)	C3—C4—H4	119.4
C1—N2—H2	116.6	C5—C4—H4	119.4
N1—N2—H2	123.2	C6—C5—C4	118.6 (4)
C1—N3—H3B	121.8	C6—C5—H5	120.7
C1—N3—H3A	115.4	C4—C5—H5	120.7
H3B—N3—H3A	122.7	C7—C6—C5	121.5 (4)
C1—N4—H4B	122.8	C7—C6—Cl1	119.7 (3)
C1—N4—H4A	116.1	C5—C6—Cl1	118.8 (4)
H4B—N4—H4A	121.1	C6—C7—C8	119.5 (4)
N3—C1—N4	120.9 (4)	C6—C7—H7	120.2
N3—C1—N2	121.0 (4)	C8—C7—H7	120.2
N4—C1—N2	118.2 (4)	C7—C8—C3	120.3 (4)
O1—C2—N1	122.0 (4)	C7—C8—H8	119.8
O1—C2—C3	121.0 (4)	C3—C8—H8	119.8
N1—C2—C3	117.1 (4)	H1W—O1W—H2W	104.3
C4—C3—C8	118.9 (4)		
			
C2—N1—N2—C1	94.5 (4)	C2—C3—C4—C5	175.8 (4)
N1—N2—C1—N3	8.8 (6)	C3—C4—C5—C6	−0.8 (8)
N1—N2—C1—N4	−171.3 (3)	C4—C5—C6—C7	1.1 (8)
N2—N1—C2—O1	−15.4 (6)	C4—C5—C6—Cl1	−178.3 (4)
N2—N1—C2—C3	164.5 (3)	C5—C6—C7—C8	−1.1 (8)
O1—C2—C3—C4	−15.6 (6)	Cl1—C6—C7—C8	178.3 (4)
N1—C2—C3—C4	164.5 (4)	C6—C7—C8—C3	0.8 (8)
O1—C2—C3—C8	159.5 (4)	C4—C3—C8—C7	−0.5 (7)
N1—C2—C3—C8	−20.5 (6)	C2—C3—C8—C7	−175.5 (4)
C8—C3—C4—C5	0.5 (7)		

### Hydrogen-bond geometry (Å, °)

**Table:**

*D*—H···*A*	*D*—H	H···*A*	*D*···*A*	*D*—H···*A*
N1—H1···Cl2^i^	0.90	2.36	3.194 (4)	154
N2—H2···O1W	0.90	2.24	3.031 (4)	146
N2—H2···Cl2^ii^	0.90	2.71	3.260 (4)	121
N3—H3B···O1^iii^	0.90	1.96	2.848 (3)	167
N3—H3A···Cl2^iv^	0.90	2.43	3.280 (4)	157
N4—H4B···O1W	0.90	2.04	2.834 (4)	147
N4—H4A···Cl2^iv^	0.90	2.44	3.286 (4)	156
O1W—H1W···Cl2^v^	0.85	2.58	3.292 (4)	142
O1W—H2W···Cl2	0.85	2.30	3.134 (4)	164

Symmetry codes: (i) −*x*+1, *y*+1/2, −*z*+1/2;  (ii) −*x*+1, *y*−1/2, −*z*+1/2; (iii) *x*, *y*+1, *z*; (iv) −*x*+1, −*y*+2, −*z*; (v) *x*, *y*−1, *z*.

## References

[bb1] Bharatam, P. V., Iqbal, P., Malde, A. & Tiwari, R. (2004). *J. Phys. Chem. A*, **108**, 10509–10517.

[bb2] Bruker (2004). *APEX2*, *SADABS* and *SAINT* Bruker AXS Inc., Madison, Wisconsin, USA.

[bb3] Bruker (2005). *XPREP* Bruker AXS Inc., Madison, Wisconsin, USA.

[bb4] Dolzhenko, A. V., Pastorin, G., Dolzhenko, A. V. & Chui, W.-K. (2009). *Tetrahedron Lett.* **50**, 2124–2128.

[bb5] Grinstein, V. & Chipen, G. I. (1961). *Zh. Obshch. Khim.* **31**, 886–890.

[bb6] Hammerl, A., Hiskey, M. A., Holl, G., Klapötke, T. M., Polborn, K., Stierstorfer, J. & Weigand, J. (2005). *Chem. Mater.* **17**, 3784–3793.

[bb7] Kolev, T. & Petrova, R. (2003). *Acta Cryst.* E**59**, o447–o449.

[bb8] Koskinen, M., Mutikainen, I., Tilus, P., Pelttari, E., Korvela, M. & Elo, H. (1997). *Monatsh. Chem.* **128**, 767–775.

[bb9] Macháčková, Z., Němec, I., Teubner, K., Němec, P., Vaněk, P. & Mička, Z. (2007). *J. Mol. Struct.* **832**, 101–107.

[bb10] Macrae, C. F., Edgington, P. R., McCabe, P., Pidcock, E., Shields, G. P., Taylor, R., Towler, M. & van de Streek, J. (2006). *J. Appl. Cryst.* **39**, 453–457.

[bb11] Murugavel, S., Ganesh, G., Subbiah Pandi, A., Govindarajan, S. & Selvakumar, R. (2009*a*). *Acta Cryst.* E**65**, o548. 10.1107/S1600536809004553 PMC2968477 21582207

[bb12] Murugavel, S., Kannan, P. S., Subbiah Pandi, A., Govindarajan, S. & Selvakumar, R. (2009*b*). *Acta Cryst.* E**65**, o454. 10.1107/S1600536809003626 PMC2968605 21582126

[bb13] Sheldrick, G. M. (2008). *Acta Cryst.* A**64**, 112–122. 10.1107/S0108767307043930 18156677

[bb14] Spek, A. L. (2009). *Acta Cryst.* D**65**, 148–155. 10.1107/S090744490804362X PMC2631630 19171970

[bb15] Westrip, S. P. (2010). *publCIF. *In preparation.

